# From the groin to the brain: a transfemoral path to blood-brain barrier opening

**DOI:** 10.18632/oncotarget.28414

**Published:** 2023-05-04

**Authors:** Thomas C. Chen, Weijun Wang, Axel H. Schönthal

**Keywords:** brain metastases, glioma, NEO100, perillyl alcohol, transfemoral cannulation

## INTRODUCTION

Brain-localized diseases are particularly difficult to treat because the blood-brain barrier (BBB) prevents the vast majority of therapeutics from effectively entering the brain parenchyma and reaching their targets at sufficiently high concentrations. This is particularly true for malignant brain cancers, which include near-incurable primary cancers such as glioblastoma, but also the far more prevalent and often deadly brain metastases derived from systemic tumors of the lung, breast, skin, and other organs. In these cases, intravenous or oral drugs only sub-optimally, if at all, penetrate the BBB *en route* to their intracranial target. While it has been recognized that tumor tissue in the brain might harbor a compromised BBB (also called the blood-tumor barrier [1]) that allows conventional chemotherapeutics to achieve occasional therapeutic responses, this “leakiness” is inconsistent and in most cases insufficient to support effective therapeutic access to malignant brain lesions [2–4]. It is believed that procedures to open the BBB in a controlled and safe fashion might provide tremendous advantages by allowing optimal brain entry of any and all circulating therapeutics. One could predict that therapeutic agents would be enabled to unfold their beneficial activities as much against cerebral lesion sites as they do against their peripheral targets, and malignancies of the brain would no longer be shielded inside their formerly BBB-protected sanctuary.

For the past several decades, intracarotid injection of hyperosmolar mannitol has been applied as a method to open the BBB [5]. This procedure has indeed demonstrated some benefit in cases of methotrexate chemotherapy for primary central nervous system lymphoma (PCNSL) [6]. However, the mannitol-based method also harbors significant risks, including seizures, brain embolisms and renal failure, and therefore its application remains restricted to the settings of well-equipped medical centers [7, 8]. A newer procedure, magnetic resonance imaging (MRI)-guided pulsed focused ultrasound (pFUS) in combination with intravascular microbubbles, has advanced to the clinic, but is limited by skull penetration of the ultrasonic waves [9].

Devising yet another promising strategy aimed at safe BBB opening, Wang et al. in a series of preclinical studies [10–12] introduced the novel concept of intraarterial (IA) injection of NEO100. NEO100 (NeOnc Technologies, Inc., Los Angeles, CA, USA) is an ultra-pure, pharmaceutical-grade version of perillyl alcohol, a naturally occurring monoterpenoid that is present, for example, in the essential oils of citrus fruit peel as a metabolite of limonene. Like perillyl alcohol [13], NEO100 has been extensively investigated for its cancer therapeutic potential, and recently published results from a Phase 1 trial applying intranasal delivery of NEO100 to recurrent glioblastoma patients showed exceptional safety and encouraging signs of activity [14, 15]. Independent from these intranasal studies that focused on NEO100’s potential as a cancer therapeutic, Wang et al. pursued the question whether IA injection of NEO100 would result in BBB opening in a manner that is safe and reversible, and potentially useful to enable beneficial brain entry of otherwise low-BBB-permeable therapeutics, such as methotrexate and therapeutic antibodies.

To set up their experimental system and provide initial proof of concept, Wang et al. performed IA injections in the form of ultrasound-guided intracardiac injection of a single bolus of NEO100 (0.1–5.0% in saline solution) into the left ventricle. Immediately following this procedure, the mice received a quick intravenous (IV) injection of Evans blue (EB), a commonly used dye with very high affinity for serum albumin. Because of its tight binding to these rather large globular proteins (about 65,000 Daltons), EB is unable to penetrate an intact BBB [16]. However, in cases of compromised or experimentally opened barrier function, the EB-protein complexes can enter the brain and effectively stain the brain tissue blue. This effect is exactly what Wang et al. observed: after IA injection of NEO100 in combination with IV EB, the euthanized mice presented with blue brains [12]. Intriguingly, this BBB-opening effect could not be achieved with similar injections of mannitol, indicating that the mechanism of BBB opening by NEO100 was different from the one exerted by hyperosmotic mannitol. However, accompanying *in vitro* and electron microscopy studies did suggest that disruption of tight junction linkages played a prominent role in NEO100’s effect [12].

Further experiments used methotrexate as a representative non-BBB-permeable chemotherapeutic agent, as well as labeled antibodies, which also are known to minimally penetrate an intact BBB. In all instances, IA injections of NEO100 resulted in substantially enhanced brain entry of the tested compounds [12]. Additional analyses established that NEO100-mediated BBB opening persisted for 2–4 hours, after which time the barrier function of the BBB fully recovered. It was also confirmed that IV injections of NEO100 were unable to achieve this BBB-opening effect. In all, the procedure appeared reasonably well tolerated, as mice subjected to it recovered promptly and continued to thrive.

The main question that followed was whether BBB opening by IA NEO100 would be able to achieve a therapeutic advantage for the treatment of brain tumors. To provide this answer, Wang et al. utilized mice with intracranially implanted tumor cells as their experimental system. In one approach [10], they used breast cancer cells engineered to overexpress human epidermal growth factor receptor 2 (HER2), which served as a xenograft model of brain-metastatic breast cancer; the anti-HER2 antibody trastuzumab was selected as the therapeutic agent. In another approach [11], they used syngeneic models of brain-metastatic melanoma and glioblastoma, where the latter served as a representation of a primary brain cancer type; immune checkpoint-inhibitory antibodies were selected as the therapeutics of choice.

Mice with intracranial HER2+ breast cancer cells received a single IA injection of 0.3% NEO100 for BBB opening, which was immediately followed by a single IV injection of trastuzumab. Comparison groups of mice received IV trastuzumab without prior IA NEO100, or no treatment at all. A few mice from each group were euthanized 24 hours later and subjected to immunohistological analysis. Their brains showed the prominent presence of trastuzumab within the tumor region, but only in those cases where IV trastuzumab had been combined with IA NEO100. Trastuzumab was not detected in brains from animals that had received trastuzumab without prior NEO100, confirming that IA NEO100 was required to enable trastuzumab to reach the brain tumor [10]. Those mice not subjected to histological brain analysis were let survive and were observed over time. Final analysis of their survival showed that untreated animals (*n* = 6) all succumbed to disease within 30 days; mice receiving IV trastuzumab without IA NEO100 (*n* = 6) died within a similar timeframe, although one animal survived until Day 66. In comparison, mice treated with IV trastuzumab secondary to IA NEO100 (*n* = 7) survived much longer, with 4 animals (57%) still remaining alive at the end of the observation period on Day 80 (*p* < 0.0001). A similar experiment, using the trastuzumab-drug conjugate ado-trastuzumab emtansine (T-DM1), yielded comparable results: IV injection of T-DM1 alone did not extend survival, but when preceded by IA NEO100, survival of all treated mice was significantly prolonged (*p* < 0.007) [10].

In the next scenario [11], Wang et al. intracranially implanted immunocompetent mice with murine glioblastoma or highly metastatic murine melanoma cells. Once tumors were established, what followed was a single injection with antibodies targeting either programmed cell death protein 1 (PD-1) or programmed death-ligand 1 (PD-L1), with or without prior BBB opening via IA NEO100. Similar to the results obtained with the above-described model of brain-metastatic breast cancer and trastuzumab, a single IV injection of checkpoint-inhibitory antibodies did not result in significant therapeutic activity. However, when any of these injections were preceded by IA NEO100, there was a striking survival benefit, where in some instances 100% of treated mice (*n* = 6 per experiment) survived until the endpoint of 150 and 300 days, respectively, which the authors viewed as a potentially curative effect [11]. Considering the highly aggressive nature of the cell lines chosen for these experiments, i.e., GL261 glioblastoma and metastatic B16F10 melanoma, these results are extraordinary and generate high hopes for future clinical testing.

While these studies may open new horizons for optimized drug delivery in cases of CNS diseases, certain limitations were noted, which will need to be investigated. On one hand, these preclinical models achieved their impressive therapeutic outcomes by applying a single IA injection of NEO100 combined with a single IV injection of therapeutic agent (or therapeutic agent combined with NEO100 in a single IA injectate). On the other hand, it remains uncertain whether such single applications may suffice to achieve similarly impressive therapeutic outcomes in the clinical setting. It is conceivable that multiple interventions might become necessary, especially in light of the heterogeneity of many tumors [17], which might be more complex than a relatively homogeneous cell line used *in vivo*. Similarly relevant is the question of appropriate translation of the IA procedure from experimental rodents to human patients. Clearly, IA injections performed as ultrasound-guided intracardiac injections—which represent the appropriate IA approach in mice and is necessitated based on their small body size—would not be suitable for humans. Addressing this issue, Wang et al. propose [11] that transfemoral arterial cannulation with fluoroscopy-guided threading of the catheter to the cranial arteries could serve as the equivalent clinical method for the controlled and safe IA injection of NEO100 in patients.

**Figure 1:**
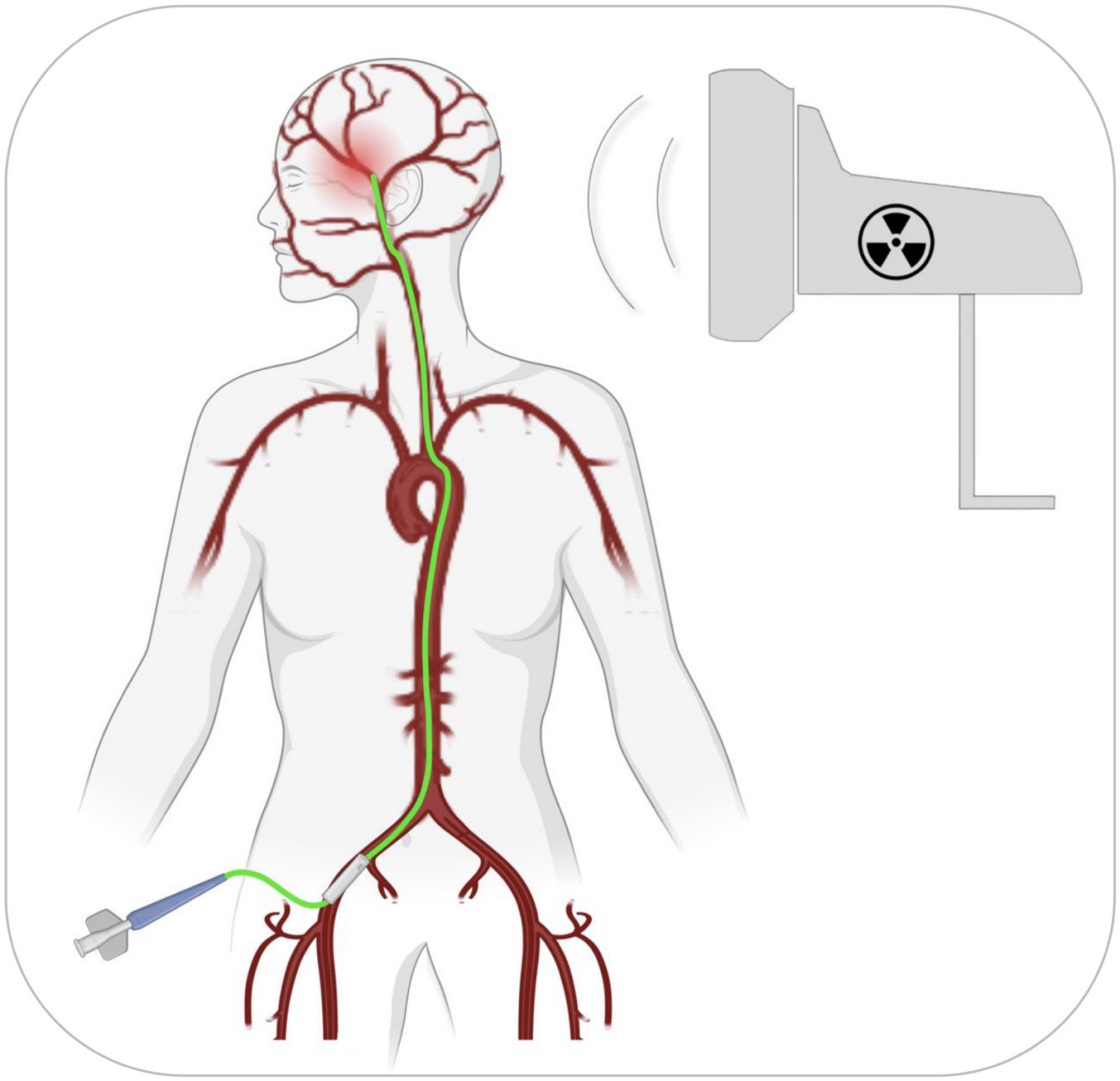
A transfemoral path to BBB opening. After access to the femoral artery has been established, the catheter (shown in green color) enters the femoral artery with advancement to the iliac artery, up the abdominal aorta, through the aortic arch to one of the carotid arteries, all the way to one of the cerebral arteries closest to the area of the malignant lesion (shown as diffuse red area). Advancement of the catheter can be visually monitored and guided by fluoroscopy (top right: fluoroscopic equipment). Telescoping catheters may be used, where the largest and outermost goes from the femoral artery to the carotid artery in the neck; through this, a smaller and longer catheter is advanced to the base of the skull, and finally, the innermost catheter, the longest and smallest, is advanced to the brain artery of interest.

Transfemoral IA catherization (Figure 1) is a low-risk procedure that is routinely performed by endovascular neurosurgeons in the context of cerebral angiograms, aneurysm coiling, tumor embolization, and thrombectomies [18]. It is considered “the gold standard technique for catheter-based neuro-interventions” [19]. However, it has never been used as a means to access tumor-feeding cranial arteries for purposes of BBB opening. In conjunction with NEO100 as the injectate, it therefore would represent a principally novel application of this method. Based on its relative safety and uncomplicated nature, it is conceivable that repeat applications might be feasible, if indeed required for the most aggressive and heterogenous tumor types. The authors envision that clinical implementation of this new BBB-opening method might achieve a similarly high rate of success in the treatment of brain-localized malignancies as do current treatments for peripherally distributed tumors; as a result, reduced morbidity and increased patient survival is expected.
